# Implementation of high‐cadence cycling for Parkinson’s disease in the community setting: A pragmatic feasibility study

**DOI:** 10.1002/brb3.2053

**Published:** 2021-02-09

**Authors:** Kathleen E. McKee, Remy K. Johnson, James Chan, Anne‐Marie Wills

**Affiliations:** ^1^ Department of Neurology Massachusetts General Hospital Boston MA USA; ^2^ Henry and Allison McCance Center for Brain Health Massachusetts General Hospital Boston MA USA; ^3^ Harvard Medical School Boston MA USA; ^4^ Department of Biostatistics Massachusetts General Hospital Boston MA USA; ^5^ Present address: Neurosciences Clinical Program Intermountain Healthcare Murray UT USA

**Keywords:** bicycling, exercise, feasibility, Parkinson's disease

## Abstract

**Background:**

Efficacy of exercise to improve motor symptoms in Parkinson's Disease (PD) has been established in multiple clinical trials. The Pedaling for Parkinson's ™ (PFP) program is an existing community‐based cycling intervention for individuals with PD. Although PFP program design was informed by in‐laboratory efficacy studies, the implementation and effectiveness of the program in the community have not been studied. This feasibility study explores implementation and effectiveness of PFP utilizing the RE‐AIM implementation evaluation framework.

**Methods:**

This was a pragmatic open‐label multi‐site study. First, community‐based gyms were recruited to implement the PFP protocol with enhanced multi‐modal training and support. Second individuals with Hoehn and Yahr stage I‐III idiopathic PD were recruited to participate. Reach, effectiveness (both clinical scores and participant enjoyment), adoption, implementation (gym and participant fidelity, cost), and maintenance (sustainability) were assessed. Tracking of adverse events was used to monitor safety of the intervention.

**Results:**

Reach was moderate: 59% of participants who expressed interest opted to participate. No effectiveness outcomes demonstrated a significant change from pre to post; however, the program was highly enjoyable (96% of participants who started classes enjoyed the program and 87% wished to continue). Adoption was poor with only four out of 34 gyms participating. The program had poor gym and moderate participant fidelity. The program was maintained for at least 4 months across all sites. The program was implemented safely.

**Conclusion:**

Barriers to implementation of nonpharmacologic interventions such as exercise protocols limit reach and availability of these interventions to patients. Pilot studies are needed to inform and direct further implementation efforts. Our pilot study suggests the PFP cycling intervention should be modified prior to attempts at widespread implementation. Modifications made by gyms in this study suggest adaptations to the protocol that may increase fidelity and effectiveness.

## Graphical abstract

This feasibility study explores implementation and effectiveness of an existing community‐based high‐cadence cycling protocol for Parkinson's disease utilizing the RE‐AIM implementation evaluation framework. Findings suggest the cycling intervention should be modified prior to attempts at widespread implementation. Modifications made by gyms in this study suggest adaptations to the protocol that may increase fidelity and effectiveness.

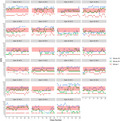

## 1 INTRODUCTION

Parkinson's Disease (PD) is now the fastest‐growing neurologic disorder in the world (Dorsey et al., 2018). Rapid translation of novel therapies into clinical practice is urgently needed to combat the growing burden of disease. Unfortunately, challenges inherent in implementation slow the rate at which proven treatments become accessible to patients (Rapport et al., 2018). Pilot studies are necessary to evaluate whether an intervention is appropriate for the cost and resources necessary to proceed with a randomized controlled implementation trial (Eldridge et al., 2016).

In PD, progressive rigidity, tremor, slowness, and falls, along with myriad nonmotor symptoms rob patients of quality of life (Dorsey & Bloem, 2018). Pharmacologic therapy mitigates symptoms but has not been shown to protect the brain from further damage and degeneration (Ahlskog, 2010; Fox et al., 2018). Nonpharmacologic therapy—in particular exercise—also improves symptoms and may even slow disease progression and protect neurons (Ahlskog, 2011, 2018; Schenkman et al., 2018).

Exercise currently dominates the field of Parkinson's research: The largest number of new randomized controlled trials for PD are exercise based. Additionally, current society guidelines recommend exercise as a “clinically useful” strategy for persons with Parkinson's (Fox et al., 2018). Many exercises such as dance (McKee & Hackney, 2013), tai‐chi (Hackney & Earhart, 2008; Li et al., 2012), running (Schenkman et al., 2018), boxing (Combs et al., 2011, 2013), Nordic walking (Monteiro et al., 2017; van Eijkeren et al., 2008), qigong (Schmitz‐Hubsch et al., 2006), aquatic exercise (Kurt et al., 2018; Perez‐de la Cruz et al., 2016), and bicycling (Ridgel et al., 2009, 2015) have demonstrated efficacy in small clinical trials.

For patients to benefit from exercise, however, protocols developed and executed through clinical trials must be adopted for use in community settings where patients can access them. Exercise protocols, in particular, can be challenging to implement with fidelity because protocols, equipment, and duration of interventions are often modified in the transition from laboratory to community setting. While these modifications are sometimes necessary due to logistical limitations in a community setting, they may also happen inadvertently by community organizations not versed in rigorous protocol implementation. The very process of community‐based implementation can also ideally further inform efficacy trials: there is little utility in perfecting an exercise regimen for PD in the laboratory if it simply cannot be implemented in the real world.

We designed a pilot study to explore implementation and clinical effectiveness of a forced high‐cadence cycling paradigm adopted for a real‐world setting. The RE‐AIM framework (Glasgow et al., 1999) was used to assess the implementation. RE‐AIM includes five domains: (a) Reach, or the proportion of the target patient population that receives the intervention; (b) Effectiveness—defined here as clinical effectiveness of the intervention; (c) Adoption, or the proportion of sites or individuals in sites willing to initiate the intervention; (d) Implementation, or cost of and fidelity to the implementation strategies; and (e) Maintenance, or the sustainability of the intervention with time. Safety was also evaluated through tracking of adverse events.

### 1.1 Why cycling?

We chose to study cycling for three reasons. First, cycling may be especially beneficial because it is often inexplicably but remarkably preserved in individuals with advanced PD who would never be able to run or complete many of the other exercise interventions (Snijders & Bloem, 2010; Snijders et al., 2012). Second, already in existence is a community‐based adaptation of forced high‐cadence cycling for PD: the Pedaling for Parkinson's™ (PFP) program (Alberts, 2019). Third, this program has not been studied in the community, and to our knowledge, there are no published studies on the implementation or effectiveness of cycling programs for PD in a community setting.

### 1.2 Current evidence for efficacy of cycling for PD

Multiple small controlled trials have demonstrated that forced high cadence cycling (FHCC) can ameliorate motor symptoms in individuals with PD as measured by the Unified Parkinson's Disease Rating Scale‐Part III and Timed up and Go (Alberts et al., 2011; Beall et al., 2013; Harper et al., 2019; McGough et al., 2016; Miller Koop et al., 2019; Ridgel et al., 2009, 2011, 2013, 2015; Uygur et al., 2015). In FHCC, individuals with PD pedal with either a tandem corider or a motor providing external augmentation at a cadence of 80–90 revolutions per minute (rpm)—which is faster than most individuals would pedal on their own. Although the rate is augmented, cycling on either device is an active, not passive, activity. In comparing FHCC versus cycling at a self‐selected cadence on a stationary indoor bicycle, most of the published literature has found that, despite similar cardiovascular exertion in the two modes, improved motor symptoms are only observed in the forced‐cadence modality (Harper et al., 2019; Ridgel et al., 2009, 2015; Uygur et al., 2015). One recent study did demonstrate gains in both forced and voluntary groups, but it was noted that the voluntary group self‐selected to pedal at a cadence near the target achieved by the forced group (Miller Koop et al., 2019).

### 1.3 Adaptations for implementation in the community

Despite the noted benefit of FHCC, the tandem or motor‐augmented bicycle equipment is not readily available or affordable. Based on his research on FHCC at The Cleveland Clinic, Jay Alberts, PhD along with Cathy Frazier, a person with Parkinson's, launched The Pedaling for Parkinson's™ (PFP) program (Alberts, 2019), as an accessible and affordable alternative to FHCC. In PFP, individuals with PD are verbally coached to achieve moderate‐exertion, high‐cadence cycling (HCC) on solo‐rider “spin” bikes. This differs from FHCC because there is no physical augmentation, only auditory and social cues encouraging participants to pedal at a high rate.

Although approximately 100 gyms across the country have adopted the PFP program, effectiveness of the program has not been established (Alberts, 2019). Written instructions on how to run the PFP program are available; however, per author (KEM) correspondence with gyms that had already implemented a Parkinson's cycling program: most gyms sought out additional help in starting their program from PFP leadership, a knowledgeable participant, or from a for‐profit company that charged a fee. Based on this information, we hypothesized that community gyms might be more willing to implement the program within their existing infrastructure and without prior specialized knowledge of PD if they had additional support. We therefore developed a pilot study to test an implementation strategy of enhanced multi‐modal training coupled with ongoing local support to supplement the existing written PFP start‐up materials.

### 1.4 Aims and hypothesis

We designed a pragmatic feasibility study to explore implementation and effectiveness of PFP in the community. We hypothesized that PFP HCC could be implemented with high gym fidelity and participant adherence in a community‐based setting and would be affordable, sustainable, and safe. If such implementation was achieved, we hypothesized HCC would be effective—that is, would lead to motor, cognitive, and quality of life gains similar to those observed in controlled trials utilizing FHCC, and that participants would enjoy the program.

## 2 METHODS

### 2.1 Study design

The feasibility study was prospectively registered on ClinicalTrials.gov, Identifier: NCT03675932. Primary implementation outcomes were initially specified as safety and tolerability. Secondary effectiveness outcomes initially included measures of motor severity (MDS‐UPDRS III, TUG), cognition (Trails A&B), and quality of life (PROMIS sf v1.0) with the intention of measuring against a historical control group. Assessment of intelligibility of dysarthria was also evaluated with a priori plans to report this speech effectiveness outcome in a separate paper. Deviation from the registered design was deemed necessary when statistical analysis determined a historical control group would not be possible. All measures of motor severity and intelligibility of dysarthria were still collected but were not measured against a historical control group. Additionally, during implementation efforts in this pilot trial, we expanded implementation outcomes to cover the full scope of the RE‐AIM framework. We felt this data would be more comprehensive and easier to interpret with the use of such a framework.

Pre‐to‐post outcomes were measured within 2 weeks pre‐ and 1‐week post participation in the classes. All other outcomes were measured as specified below. The protocol and consent forms were approved by the Partners Human Research Committee. All procedures followed were in accordance with the ethical standards of the responsible institutional or regional committee on human experimentation or in accordance with the Helsinki Declaration of 1975, as revised in 1983. All participants provided written informed consent. Study data were collected and managed using REDCap electronic data capture tools hosted at Partners Healthcare (Harris et al., 2009).

### 2.2 Implementation strategy

We recruited gyms in the greater Boston area to implement PFP classes based on their geographic diversity and access to participants with PD. As per PFP protocol, gyms were required to read and sign the PFP licensing agreement. This agreement details general instructions for implementation of the program along with legal requirements for participation. In order to encourage the adoption of the program, we designed an augmented implementation strategy which included the following components: first, staff at each site underwent an in person or by phone 45‐min protocol training. Second, staff attested in writing to viewing a 60‐min video produced by study investigators (KEM) that (a) introduced the clinical features of PD (b) summarized the research behind FHCC (c) detailed how to set up a PFP class (McKee, 2018). Third, gyms were provided with premade handouts detailing the structure of the classes, highlighting exertion targets, and how to record participant bike settings. Fourth, gyms received ongoing study staff support with the opportunity to ask questions regarding implementation.

### 2.3 Intervention

Similar to several FHCC studies (McGough et al., 2016; Miller Koop et al., 2019; Ridgel et al., 2009), the duration of the intervention was 24 one‐hour spin classes over 8–9 weeks. The PFP protocol consists of a 10‐min warm‐up, 40‐min main set, and 10‐min cooldown. During the main set, participants target, a cadence of 80–90 rpm and an exertion level of either 60%–80% of their maximum heart rate or between 4 and 7/10 (somewhat hard to very hard) on the Borg category ratio rating of perceived exertion scale (Borg CR10), a 0–10 scale that has been validated in PD (Borg, 1973; Penko et al., 2017). If participants cannot achieve the full protocol, instructors encourage rest breaks to allow safe maximal participation. After the intervention concluded, gyms and participants decided independently if they would continue offering/taking classes.

### 2.4 Study participants

Participants were recruited through flyers, referral, PD support groups, websites, and a targeted approach whereby health system participants who had already consented to be contacted for research purposes were identified by zip codes proximal to gym locations. Participants could choose their preferred gym from the list of participating gyms. Some participants were pre‐existing members of the gym at which they chose to participate. Full eligibility criteria are detailed in Appendix S1. Participants had a clinically confirmed diagnosis of Hoehn and Yahr stage I‐III idiopathic PD while ON antiparkinsonian medication, and stable medication regimen. Participants agreed not to initiate a new structured exercise plan or new course of physical therapy for the duration of the intervention but could continue any pre‐existing exercise routine (including group classes).

At the first visit, participants were asked to provide demographic data, medical history, a list of their PD medications, frequency of falls in the week prior, and information about prior exercise experience. They also underwent measures of physical activity and cognition via International Physical Activity Questionnaire‐Short Form (Craig et al., 2003) and the Montreal Cognitive Assessment (Chou et al., 2010). These instruments were not used as outcome measures but rather to help characterize the population who chose to participate in the intervention.

### 2.5 Implementation framework

We used the RE‐AIM implementation science framework to evaluate implementation and effectiveness of the intervention (Table 1).

**TABLE 1:** RE‐AIM framework to evaluate implementation of pedaling for Parkinson's™

Domain	Definition	Study population(s)	Study measures
Reach	The proportion of the targeted population that receives the intervention	Participants	# participated/# screened for participation
Effectiveness	Clinical effectiveness of the intervention	Participants	MDS‐UPDRS III^a^ (motor outcome) TUG^b^ (motor outcome) TRAILS A&B^c^ (motor & cognitive outcome) PROMIS sf v1.0^d^ (quality of life outcome)
	Acceptability/enjoyment of the intervention	Participants	Likert scale & free‐text questions
		Gyms	Likert scale & free‐text questions
Adoption	The proportion of sites or individuals in sites willing to initiate the intervention	Gyms	# gyms who implemented the program/# of potential participating gyms
Implementation	Fidelity to the implementation strategies	Gyms‐fidelity	Direct observation of classes Cadence Rating of perceived exertion
		Participants‐fidelity (adherence)	# who did not withdraw from the study, were not lost to follow up, and completed at least 80% of the sessions/# who started study
	Cost of the implementation strategy	Cost‐implementation	Estimated based on study staff record of implementation strategy cost
		Cost‐participants	Estimated based on a post study survey querying gyms and participants about incurred costs
Maintenance	Sustainability of the intervention with time	Gyms	# offering classes 8 weeks post/# who participated in study
		Participants	# participating in HCC 8 weeks post/# who participated in study

^a^ MDS‐UPDRS III: Movement Disorder Society—Unified Parkinson's Disease Rating Scale‐Part III.

^b^ TUG: Timed Up and Go.

^c^ TRAILS A & B: Trail Making Test, Parts A & B.

^d^ PROMIS: Participant‐Reported Outcomes Measurement Information System.

*Reach* was narrowly defined as the percentage of people who decided to participate out of those screened. Although this captures reach of the program only among participants who were aware of and interested in it, we felt it was an important measurement because a low reach may indicate barriers to participation in the community which would need to be more fully investigated prior to a randomized controlled trial of implementation.

*Effectiveness* was defined as both clinical effectiveness of the intervention as well as acceptability/enjoyment of the intervention by participants and gyms. For clinical effectiveness outcomes, participants were evaluated within 2 weeks before (pre‐test) and 1 week after (post‐test) the intervention. Participants were tested “ON” medications at the same time of day (within 1 hr) to reduce medication‐related performance fluctuation. Post‐test evaluations were not conducted on the last day of class due to previously documented motor and cognitive improvements immediately following a single session of FHCC (Ridgel et al., 2013). A modified version of the Unified Parkinson's Disease Rating Scale UPDRS; Fahn & Elton, 1987; excluding rigidity and retropulsion was used so that it could be videotaped and rated remotely by a movement disorders neurologist blinded to whether the visit was pre‐ or postintervention. This modified version has been shown to be reliable and valid, both at cross‐sectional time points and longitudinally (Abdolahi et al., 2013). Timed Up and Go (TUG; Shumway‐Cook et al., 2000) and Trail Making Test (TMT) A & B (Lezak, 1995; Reitan & Wolfson, 1993) were tested. Quality of life was assessed via PROMIS‐Global Health v1.1: A 10‐item questionnaire that assesses participant‐reported outcomes regarding their overall (global) health (Cella et al., 2010; Hays et al., 2009). This instrument produces physical and mental health T‐scores, the distributions of which are standardized such that a 50 represents the mean for the US general population and the standard deviation around that mean is 10 points. This instrument was chosen as a more global assessment than the Parkinson's Disease Questionaire‐39 (PDQ‐39). Acceptability/enjoyment of the intervention was measured through Likert scale (McKee & Hackney, 2013) and free‐text questions designed to capture participant and gym subjective experience.

*Adoption* of the PFP program was measured by the number of potential participating gyms with spin bikes who ultimately implemented the program.

*Implementation* was measured through fidelity and cost outcomes. *Fidelity* of *gyms* to PFP protocol was measured via direct observation of classes by author KEM in which actual class structure was compared to the structure specified in the written PFP licensing agreement, as well as each gym's subjective report of whether they could implement the class as per protocol. Additionally, in‐class cadence and RPE data were collected by spin instructors to determine whether gyms were generally meeting cadence and exertion targets with their participants. Instructors had participants rate their exertion using the Borg CR10 RPE scale at 1, 20, and 39‐min into the main set. Instructors simultaneously recorded participant cadence at these time points through spot‐check of the bike's cadence monitor. Average cadence and exertion were measured in this way because community‐based classes often lack access to continuous cadence and heart rate monitoring. *Participant fidelity or adherence* to the intervention was measured through record of attendance over the 24 offered sessions. We considered those who started the classes, did not withdraw from the study, were not lost to follow‐up, and completed at least 80% of the sessions to have adhered to the intervention. *Cost* was estimated based on (a) study staff record of implementation strategy cost and (b) a poststudy survey querying gyms and participants about intervention costs they incurred.

*Maintenance* was measured via telephone 8 weeks after conclusion of the intervention to determine if gyms continued to offer PFP classes and participants continued HCC.

*Safety* was monitored through tracking of adverse events (AE) as reported by instructors. Participants were queried by phone regarding interval AE between weeks 3–6, and 8 weeks after the intervention. Serious AE were defined as life‐threatening, requiring hospitalization, or leading to a persistent disruption of baseline function.

### 2.6 Statistical analysis

Reach, effectiveness‐acceptability, adoption, maintenance, and some implementation outcomes are reported descriptively as specified in Table 1. To determine independent baseline predictors of participant fidelity or adherence (a measure of implementation), comparison of covariates across adherence was carried out using a Wilcoxon rank‐sum test for continuous variables and a Fisher exact test for categorical variables. Clinical effectiveness outcomes were analyzed on an intention to treat basis and tested against the null hypothesis of no change using a mixed‐effects linear model with a fixed effect for time (pretest or post‐test) random effects for site and participant. Clinical effectiveness outcomes aside from PROMIS global health are reported as estimated mean change in scores.

### 2.7 Sample size

If the true adherence proportion is 80%, we calculated that a sample of 30 participants would provide an estimate of the true adherence with a confidence interval of 0.31 (0.61–0.92). We also calculated that a sample size of 30 participants would provide 80% power to reject the null hypothesis of no change in UPDRS scores compared with an alternative hypothesis of 3.3 points mean change using a significance level of 5% (two‐tailed) and a standard deviation of 6.0. Previously reported data in exercise studies for PD indicate that UPDRS scores will have a standard deviation of 6.0 for the change from baseline to week 8 when measured OFF medication (Ridgel et al., 2009). We expected a smaller standard deviation since we utilized a modified version of the UPDRS and tested participants ON medication. Comparison to a hypothesized value of no change was based on previous studies that have shown no improvement in UPDRS scores for those participating in voluntary exercise (Ridgel et al., 2009, 2015).

## 3 RESULTS

Using the RE‐AIM framework, results of the study are summarized below.

### 3.1 Reach

**FIGURE 1:**
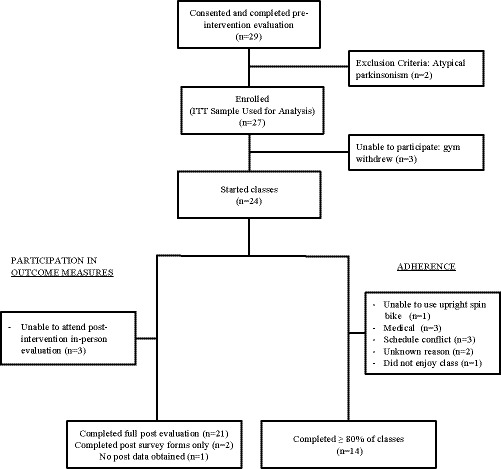
Participant Participation and Adherence. All participants who enrolled were analyzed with intention to treat (ITT) analysis. Medical reasons for nonadherence included arthritis, back pain, and complication from elective surgery

48 individuals were prescreened and confirmed eligible. 19 of those individuals declined to participate, most commonly due to inconvenient class time or location. 29 individuals were consented and completed the preintervention evaluation. Two individuals were subsequently excluded when their diagnosis was discovered to be atypical parkinsonism. Reach was 59%: 27 enrolled out of 46 eligible participants. Figure 1 depicts participant participation as well as adherence. Baseline characteristics of the 27 enrolled participants are listed in Table 2. Participants were older, highly educated white adults with moderate disease severity. At baseline, this cohort already had a high level of physical activity measured using the IPAQ‐sf. Four participants had fallen one or more times in the week prior to the baseline assessment. 93% (*n* = 25) of participants had prior experience on a road or stationary bike with 78% (*n* = 21) endorsing comfort cycling outdoors on a road or bike path. Only 17% (*n* = 4) had ever previously participated in a spin class and only one of those individuals at a frequency of three times a week or more.

**TABLE 2:** Participant characteristics

Characteristic^a^	All participant (*n* = 27)^e^	Adherence to intervention (*n* = 14)	Non‐adhere to intervention (*n* = 10)	*p* ^d^
Age (years)	68.1 (8.05)	69.4 (8.53)	65.0 (7.62)	.259
Sex (% female)	30% (8)	14% (2)	50% (5)	.085
White race, non‐Hispanic ethnicity	100% (27)	100% (14)	100% (10)	1
Bachelor's degree or higher	78% (21)	64.3% (9)	90% (9)	.341
Body mass index (kg/m^2^)	26.1 (4.45)	27.5 (3.91)	24.8 (4.89)	.122
Hoehn and Yahr stage				
I	19% (5)	14% (2)	30% (3)	.25
II	67% (18)	64% (9)	60% (6)	
III	14% (4)	21% (3)	10% (1)	
Time since PD diagnosis (years)	5.8 (5.51)	5.6 (5.54)	6.2(6.51)	.703
Duration of symptoms (years)	7.4 (5.58)	7.6 (6.19)	7.8 (5.63)	.664
Implanted deep brain stimulator	7% (2)	7% (1)	0.0% (0)	1
Levodopa equivalent daily dose (LEDD) (mg)	487.9 (439.30)	469.8 (458.49)	465.1 (271.36)	.618
Baseline Montreal Cognitive Assessment Score (/30)	25.63 (3.40)	26.0 (3.51)	24.9 (3.75)	.237
Baseline IPAQ‐sf^b^ level of physical activity				
High	56% (15)	57.1% (8)	50.0% (5)	.715
Moderate	33% (9)	35.7% (5)	20.0% (2)	
Low	11% (3)	7.1% (1)	30.0% (3)	
Baseline PROMIS^c^ global health				
Physical T‐score	48.7 (7.21)	49.3 (7.41)	46.6 (4.83)	.575
Mental T‐score	51.1 (8.39)	50.0 (7.60)	51.4 (10.14)	.368

^a^ All measures reported as Mean (*SD*) or Percent (*n*) except PROMIS is reported as T‐scores.

^b^ IPAQ‐sf: International Physical Activity Questionnaire—short form.

^c^ PROMIS: Participant‐Reported Outcomes Measurement Information System. PROMIS scores of 48.7 and 51.1 can be interpreted to mean physical and mental health of this cohort fell near the national average.

^d^ *p* values reflect comparison between characteristics of those who adhered to the intervention as compared to those who did not.

^e^ Three participants enrolled in the trial but were not able to undertake the intervention as their gym dropped out. Their data is not reflected in the adherence comparison but is reflected in the second column detailing baseline characteristics of all participants.

### 3.2 Effectiveness

Estimates and standard errors of change in clinical effectiveness outcomes from longitudinal regression models are shown in Table 3. There was no significant improvement in any of the clinical effectiveness outcomes. Acceptability and enjoyment of the program were high among gyms and participants. All four gyms “strongly agreed” that they enjoyed offering the PFP program and that it was easy to implement. Gyms agreed (“somewhat agreed” to “strongly agreed”) that participants achieved target cadence and experienced motor and cardiovascular gains. Of the 23 participants who completed the postsurvey: 96% agreed they enjoyed participating in the program and 87% agreed they would continue participating if they could. 70% agreed their mood improved; 83% agreed their endurance improved. Appendix S2 further details participant responses.

**TABLE 3:** Exploratory effectiveness outcomes

Outcome	Preintervention^a^	Estimated change (95% CI)	*SE*	*p* value
PROMIS‐global health				
Physical *t*‐score	48.15	−1.81 (−4.87–1.26)	1.53	.25
Mental *t*‐score	50.6	−1.97 (−4.96–1.01)	1.49	.2
Modified UPDRS (/84)	13.55	0.3 (−1.12–1.67)	0.69	.67
TUG (sec)	10.86	0.45 (−0.22–1.11)	0.33	.19
TMT A (sec)	41.65	1.86 (−1.45–5.11)	1.63	.27
TMT B (sec)	85.44	−8.18 (−22.08–4.87)	6.62	.23

^a^ Preintervention values are reported as mean estimates.

In free‐text response, 43% of participants listed camaraderie as something they liked best about the program while 26% cited the instructor and 22% cited the structure as favorite parts of the program. Other likes included: access, the challenge, the facility, motor benefit, music, participating in research, and “everything.” The most common dislike was saddle soreness cited by 35% of participants. 30% of participants said there was “nothing” they disliked about the program. 17% disliked traveling to participate. Selected comments made by participants during AE check‐ins are listed in Appendix S3.

### 3.3 Adoption

**FIGURE 2:**
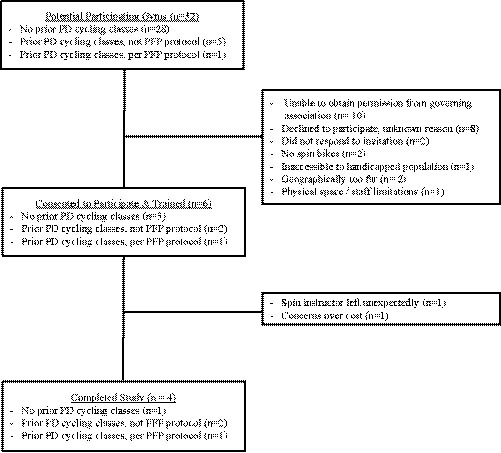
Gym Recruitment and Participation. Recruitment, retention, and participation of community‐based gyms are depicted in this diagram. 32 gyms were contacted regarding participation in the study. Four gyms (including three YMCAs) ultimately completed the study

Figure 2 describes gym recruitment and participation. 32 gyms were considered potential participants of the study, of which only six agreed to participate and completed the implementation training. Ultimately, only four gyms (including three Young Men's Christian Association (YMCA) gyms) completed the study for an adoption rate of 12%. The largest barrier to gym recruitment (10 gyms) was inability of the gyms to obtain permission of a governing association (needed to sign the licensing contract for PFP). The majority of the rest of the gyms who declined to participate (8 gyms) did not provide us with a reason for their unwillingness. Two important structural barriers identified were lack of spin bikes and lack of a handicap accessible gym entrance. Of note, only Gym A elected to start the PFP program from scratch. Gyms B and D converted their current PD cycling classes to PFP protocol; Gym C was already executing the PFP program per protocol for experienced participants but agreed to participate in the study with novel participants. Staff at all four participating gyms completed protocol training and attested to watching the training video. Gym staff reported the video was helpful and motivating, but too long.

### 3.4 Implementation‐fidelity

Gym B implemented the PFP protocol as prescribed (i.e., participants were coached to complete 10‐min warm‐up, 40‐min main set at average cadence of 80–90 rpm *for the entire time*, and 10‐min cooldown) from the start of classes. The other three gyms found the protocol too difficult for most participants to achieve and adapted the protocol to include interval training (e.g., 1‐min on, 1‐min off). Of note, although not specified in the PFP protocol, all sites chose to play music for participants during the class. Instructors reported they sought out music based on participant preference and with a tempo of 80–90 beats per minute to match that of the spin target. One site also played brain teaser, math, and “get to know you” games while cycling. These games were led by the instructor who walked around in the middle of the circle of bikes to keep participants engaged with these verbal exchanges.

**FIGURE 3:**
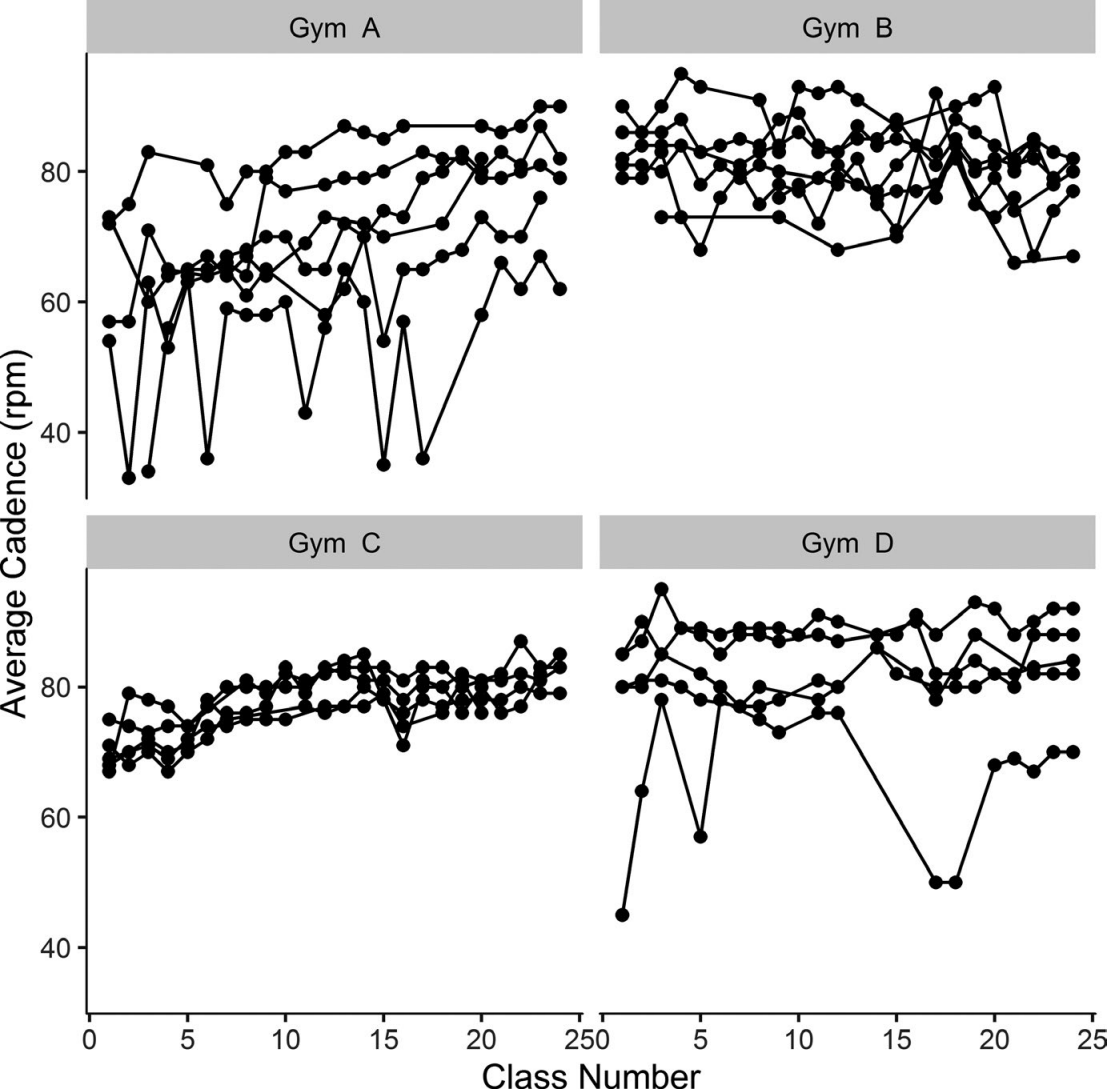
Individual Average Cadence per Class. Each individuals’ average cadence per class is graphed along with participants from the same study site

**FIGURE 4:**
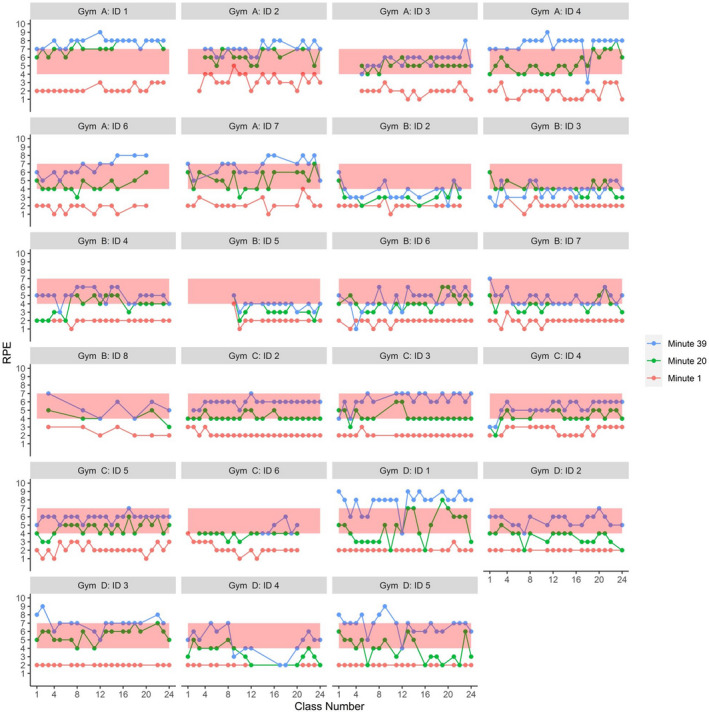
Individual Average RPE per Class. Borg CR 10 RPE at one minute into the main set is depicted in red, at 20 min in green, and at 39 min in blue. The pink bar highlights the RPE target for the duration of the main set

Average cadences are shown per participant and grouped by gym in Figure 3. Many participants in gyms C and A were consistently below cadence targets initially, but by the last week of the study, most participants at all four gyms achieved an average between 75 and 85 rpm, just shy of the 80–90 rpm target. Average per participant RPE at each of the sampled time points are shown in Figure 4. Very few participants achieved the target RPM at 1 min into the main set, and unlike cadence, RPE did not generally increase over the course of the study.

Participant fidelity (adherence) was moderate: 14 of the 24 participants (58%; 95% CI = 39% to 78%) who started classes completed at least 80% of the classes (our prespecified definition of adherence). Baseline participant characteristics did not differ significantly between those who adhered to the study and those who did not (Table 2). The most common reasons for nonadherence were medical conditions that arose over the course of the study and schedule conflicts (Figure 1). Nearly all participants traveled to class by car: 83% driving themselves and 13% driven by someone else. Average one‐way transit distance was 10.8 miles (*SD* 9.31) and time 21.9 min (*SD* 11.62). Classes were offered starting mid‐morning through early afternoon; 78% of participants found these times convenient.

### 3.5 Implementation‐cost

The cost of the implementation strategy included: cost of video production, and investigator time (~40 hr to develop video; ~12 hr supporting gyms during initial start‐up). Cost to participants varied across sites. All YMCAs chose to offer the program as an included benefit of membership. One YMCA opted to offer the program for free for the 8 weeks of the research study and thereafter charge gym membership to continue participation. One YMCA allowed nonmembers to participate for a fee of $100 for the 8‐week session. The non‐YMCA charged $11 per class.

At the conclusion of the study, participants were asked via written exit‐survey how much they would consider to be a reasonable and sustainable amount to pay for this type of program. 18 participants chose to answer and considered an average of $5.50 per class (range $0–30) to be a reasonable and sustainable amount to pay. Gyms estimated that cost to produce an 8‐week class session ranged from $1,500 to $2,200, with the majority allocated to cover salary for the instructor. Other cost components were bike lease fees, cleaning supplies, heart rate monitors, and marketing.

### 3.6 Maintenance

All four gyms were still offering the program 8 weeks after the end of the study. 18 of 23 (78%) participants who completed the postsurvey evaluation reported continuing HCC for at least some time after the end of the intervention (89% of those individuals riding at their study site). 13 of 23 (56%) participants reported cycling one or more times during the week the postsurvey was conducted (the eighth week post).

### 3.7 Safety

62% of the 24 participants who started classes reported an AE between date of consent and 8 weeks post (Table 4). Most of these AE’s were mild and not considered related to the study intervention. Pneumonia requiring hospitalization and postoperative internal bleeding after elective knee surgery were the only two serious AEs; neither was considered related to study interventions. Musculoskeletal and connective tissue disorders (primarily saddle soreness and knee pain) were the most common AEs related to the study interventions and did not affect compliance. Back pain and a broken foot after the end of the study did prevent two participants from continuing cycling after the end of the intervention. No falls occurred during or immediately before or after class but falls outside of class did limit some participants’ ability to fully participate in subsequent classes. Dyspnea and palpitations required two participants to end a single session early but did not prevent subsequent return to class.

**TABLE 4:** Frequency list of reported adverse events

Event according to system organ class or preferred term^a^	Total events *n*	Participants *n* (%)
Cardiac disorders		
Dyspnea (1)	2	2 (7%)
Palpitations (1)		
Eye disorders		
Eye hemorrhage (1)	1	1 (4%)
General disorders and administration site conditions		
Fatigue (1)	1	1 (4%)
Infections and infestations		
Pneumonia requiring hospitalization (1)	1	1 (4%)
Injury, poisoning and procedural complication		
Fall (4)	4	3 (11%)
Musculoskeletal and connective tissue disorders		
Back pain (2)	15	12 (44%)
Broken foot (1)		
Knee pain (3)		
Leg cramps (1)		
Saddle soreness (5)		
Swollen quadriceps (1)		
Shoulder pain (1)		
Plantar fasciitis (1)		
Nervous system disorders		
Hand numbness (1)	3	3 (11%)
Listing to one side on bicycle^b^ (1)		
Loss of consciousness associated with fall (1)		
Psychiatric disorders		
Depressed mood (1)	1	1 (4%)
Respiratory, thoracic, and mediastinal disorders		
Common cold (1)	4	4 (15%)
Sinus infection (2)		
Vascular disorders		
Postoperative internal bleeding (1)	1	1 (4%)

^a^ Parenthetical numbers in the first column indicate absolute number of events. Events were deemed related to study intervention if they occurred during a cycling class or appeared to be temporally related to a class (e.g., leg cramps at night but only on the nights after class). The following events were *not* thought to be related to cycling classes: eye hemorrhage, pneumonia, all falls, broken foot, back pain (1/2), knee pain (1/3), shoulder pain, loss of consciousness, all psychiatric/respiratory/vascular disorders.

^b^ Listing to one side on the bicycle may be caused by dystonia associated with PD. This phenomenon had been previously witnessed by spin instructors in another individual with PD prior to start of the study.

### 3.8 Protocol violations

Six participants violated the protocol eight times including increasing antiparkinsonian medication (*n* = 3), starting a new round of physical therapy (*n* = 4), and starting a new exercise program (*n* = 1). These participants were included in the safety, adherence, and effectiveness analyses.

## 4 DISCUSSION

This nonrandomized pilot study examined implementation of a community‐based high‐cadence cycling intervention for Parkinson's Disease utilizing the *RE‐AIM framework*. The program *reached* 59% of possible participants. Clinical *effectiveness* was not demonstrated. The PFP HCC intervention was *adopted* by only 12% of gyms with poor gym and moderate participant *implementation* (fidelity) although enjoyment of the program and *maintenance* were high. The program did appear to be safe. This study suggests intervention and implementation strategies for HCC should be modified prior to attempts at widespread dissemination. Modifications made by gyms in this study may inform future protocol adaptations to improve fidelity and effectiveness of the program.

*Gym Implementation‐fidelity* was poor with low protocol fidelity marked by a wide variety of cadences and RPE within and between sites. Only Gym B implemented the protocol as specified. We suspect Gym B was able to offer the program per protocol from the beginning because a higher proportion of their participants had prior spin class experience. The other three gyms adapted the intervention into an interval program to allow participants structured rest between attempts to achieve target cadence and exertion. Although this was in violation of the PFP protocol, these adapted interval programs resulted in most participants achieving cadence targets by the end of the 8‐week intervention.

Based on data collected from the gyms about their adaptations, Gym C developed the most robust interval program. This gym also produced the most tightly grouped participant cadences, which suggests high reliability of their program design. Future studies should examine a revised PFP protocol informed by real‐world adaptations made by these gyms. Additionally, as it took nearly the entire 8 weeks to achieve cadence goals with this adapted design, future studies should consider assessing clinical effectiveness after a longer intervention period.

*Reach* was 59% (27 enrolled out of 46 eligible) with most individuals declining to participate due to inconvenient class time or location. However, the demographics of the entirely white, highly educated, and baseline‐fit cohort limit generalizability, especially to underserved communities. For *all* patients with Parkinson's to benefit from this intervention, more research is needed to understand barriers and facilitators to diverse participant participation.

Of the enrolled participants who had access to a participating gym*, participant implementation‐fidelity* (adherence) was 58% due to medical events (mostly unrelated to study intervention) and scheduling logistics, among other reasons (Figure 1). It is unclear how this compares with other studies due to inconsistent or lack of reported adherence data (Allen et al., 2012). Future implementation attempts should focus on reducing barriers to enrollment and adherence by increasing the number of participating gyms or other options for accessing the intervention. Remote classes using Internet connected spin bikes in participant homes could be compared with in‐person classes to see whether this will improve adherence (or worsen adherence due to the lack of camaraderie).

*Adoption* by gyms was a major barrier in implementation. Out of 34 potential participating gyms, only four gyms completed the study. Three out of these four gyms already offered some form of cycling for patients with Parkinson's prior to the study; only one gym initiated the program from scratch. Failure to obtain permission from a central governing body resulted in 10 gyms’ inability to participate in the needed time frame. Barriers related to cost, equipment, and personnel were observed in a few of the gyms. Three enrolled participants were unable to complete the study because their gym dropped out over concerns of cost. Cost barriers could be addressed through partnering with philanthropic and community‐focused organizations. We found participating YMCAs were more willing to undertake the cost of setting up the programs in part because of their mission to serve the community, experience working with other chronic health conditions, and ability to subsidize programs through fundraising. Future implementation of novel exercise programming for individuals with PD may benefit from a participatory design approach which would allow earlier identification of barriers to implementation.

Despite failing to achieve success in several of the RE‐AIM measures, the PFP program was shown here to be safe, enjoyable (*effectiveness‐acceptability*), and sustainable (*maintenance*). Similar exercise programs will likely be met with enthusiasm and resources will be invested to sustain them. It is imperative to ensure programs demonstrate first efficacy and then clinical effectiveness before substantial effort is spent at widespread dissemination and implementation.

While there is evidence for the efficacy of FCC based on randomized trials conducted in controlled settings, there is no such evidence for HCC which was created as a natural modification of FCC to allow dissemination in the community. Efficacy of HCC should be established prior to attempts at wider dissemination. The HCC protocol used in such an efficacy trial should be informed by needs encountered in the real world. If efficacy is subsequently demonstrated, the protocol—having been informed by real‐world needs—may achieve effectiveness in the community. In this pilot study, the current PFP HCC protocol was not able to be implemented with high‐fidelity and did not demonstrate effectiveness. However, modifications made by gyms suggest adaptations which may improve fidelity and effectiveness. These adaptations should inform HCC protocol design for a subsequent efficacy trial.

## CONFLICTS OF INTEREST

KEM, RKJ, and JC declare they have no competing interests. AW has received research funding from the ALS Association, the Parkinson's Foundation, has participated in clinical trials funded by Acorda, Abbvie, Biogen, Bristol‐Myers Squibb, Sanofi/Genzyme, Pfizer, and received consultant payments from Acorda, Mitsubishi Tanabe Pharma and from Accordant.

## AUTHOR CONTRIBUTIONS

Kathleen E McKee: Drafting/revision of the manuscript for content, including medical writing for content; Major role in acquisition of data; Study concept or design; Analysis or interpretation of data. Remy K Johnson: Major role in acquisition of data. James Chan: Drafting/revision of the manuscript for content; Analysis or interpretation of data.Anne‐Marie Wills: Drafting/revision of the manuscript for content, including medical writing for content; Major role in acquisition of the data; Study concept or design; Analysis or interpretation of data.

### PEER REVIEW

The peer review history for this article is available at https://publons.com/publon/10.1002/brb3.2053.

[Correction added on March 20, 2021, after first online publication:Peer review history statement has been added.]

## Supporting information

Appendix S1‐S3

## Data Availability

The data that support the findings of this study are available from the corresponding author upon reasonable request (McKee et al., 2020).
