# Supplementary material for: Implementation of high‐cadence cycling for Parkinson’s disease in the community setting: A pragmatic feasibility study
Source: Brain Behav. 2021 Feb 9;11(4):e02053. doi: 10.1002/brb3.2053 (PMC8035480; doi:10.1002/brb3.2053)
Supplement: Supplementary file 1 — Appendix S1‐S3 [file BRB3-11-e02053-s001.docx]

**Supplementary Appendix 1. Study Inclusion and Exclusion Criteria**

*Inclusion Criteria*:

1. Adults age 18 or older.
2. Clinically confirmed diagnosis of idiopathic PD by a movement disorder specialist.
3. Hoehn and Yahr stage I-III while ON anti-parkinsonian medication. ^†^
4. Stable or absent MAO-B inhibitors (Selegiline, Rasagiline, Zydis selegiline HCL Oral disintegrating) for at least 60 days prior to enrollment; stable or absent other medications for PD motor symptoms (Levodopa, Dopamine agonists, Anticholinergics, Amantadine, COMT-Inhibitors) for at least 30 days prior to enrollment; stable or absent medications for the following PD-non-motor symptoms: depression, anxiety, cognition, sleep, orthostatic hypotension.
5. Agreement to defer any medication changes until after completion of eight-week program and post-test measurements.
6. Written permission by a physician to participate in the program.
7. English language proficiency sufficient to understand and participate in a cycling class taught in English.

*Exclusion Criteria:*

1. Clinically significant medical disease that would increase the risk of exercise-related complications (eg: cardiac or pulmonary disease, uncontrolled diabetes mellitus, uncontrolled hypertension or stroke) as determined by a treating physician through letter obtained by YMCA or the study investigator.
2. Dementia as evidenced by a score of less than 116 on the Mattis Dementia Rating Scale^‡^ or dementia in the opinion of the study investigator that would prohibit participant from complying with all study activities.
3. Other medical or musculoskeletal contraindication to exercise.
4. Concurrent participation in another trial of exercise therapy for PD or initiation of a new structured exercise plan during the duration of the study. Note: participants were allowed to continue any pre-existing exercise routine (including group-based classes) during the study but were asked not to start anything *new*.

^†^The Hoehn and Yahr scale is a rating scale describing the stages of PD with 1 being the least severe and 5 being the most severe. Individual patient stage can be different when assessed ON their medication vs OFF their medication.

^‡^Mattis Dementia Rating Scale is a specified as exclusion for participation in Pedaling for Parkinson’s Protocol. This criterion was utilized if a participant had a score recorded from a prior administration of scale but the scale was not collected as part of study assessments.

**Supplementary Appendix 2. Participant Experience**^†^ **of Program**

|  | **N** | **% (n)** |
| --- | --- | --- |
| **I enjoyed participating in this program.** | | |
| Strongly Agree | 23 | 87% (20) |
| Somewhat Agree | 23 | 9% (2) |
| Neither Agree nor Disagree | 23 | 0% (0) |
| Somewhat Disagree | 23 | 0% (0) |
| Strongly Disagree | 23 | 4% (1) |
| **My balance has improved.** | | |
| Strongly Agree | 22 | 18% (4) |
| Somewhat Agree | 22 | 32% (7) |
| Neither Agree nor Disagree | 22 | 40% (9) |
| Somewhat Disagree | 22 | 9% (2) |
| Strongly Disagree | 22 | 0% (0) |
| **My walking has improved.** | | |
| Strongly Agree | 23 | 17% (4) |
| Somewhat Agree | 23 | 35% (8) |
| Neither Agree nor Disagree | 23 | 39% (9) |
| Somewhat Disagree | 23 | 9% (2) |
| Strongly Disagree | 23 | 0% (0) |
| **My mood has improved.** | | |
| Strongly Agree | 23 | 39% (9) |
| Somewhat Agree | 23 | 30% (7) |
| Neither Agree nor Disagree | 23 | 22% (5) |
| Somewhat Disagree | 23 | 4% (1) |
| Strongly Disagree | 23 | 4% (1) |
| **My coordination has improved.** | | |
| Strongly Agree | 23 | 13% (3) |
| Somewhat Agree | 23 | 39% (9) |
| Neither Agree nor Disagree | 23 | 35% (8) |
| Somewhat Disagree | 23 | 13% (3) |
| Strongly Disagree | 23 | 0% (0) |
| **My strength has improved.** | | |
| Strongly Agree | 23 | 30% (7) |
| Somewhat Agree | 23 | 35% (8) |
| Neither Agree nor Disagree | 23 | 30% (7) |
| Somewhat Disagree | 23 | 0% (0) |
| Strongly Disagree | 23 | 4% (1) |
| **My endurance has improved.** | | |
| Strongly Agree | 23 | 52% (12) |
| Somewhat Agree | 23 | 30% (7) |
| Neither Agree nor Disagree | 23 | 9% (2) |
| Somewhat Disagree | 23 | 4% (1) |
| Strongly Disagree | 23 | 4% (1) |
| **If I could, I would continue participating.** | | |
| Strongly Agree | 23 | 74% (17) |
| Somewhat Agree | 23 | 13% (3) |
| Neither Agree nor Disagree | 23 | 4% (1) |
| Somewhat Disagree | 23 | 0% (0) |
| Strongly Disagree | 23 | 9% (2) |

^†^Participant experience survey is adapted from McKee and Hackney’s 2013

study of community based adapted tango dancing for PD.

**Supplementary Appendix 3. Participant Comments**^†^

| “The program was very uncomfortable at the beginning and it would have been easy to stop but [the instructor’s] encouragement/motivation were crucial. [The instructor] would do things like adjust the seat 5 times and tell us ‘I'll do is as much as your need!’ [The instructor] even cut home-made seat pads for people who needed them.”  “I felt less coordinated in the evening after having spin session because I was so tired and worn out by trying to hit the target. By the eighth week though, I felt I was improving - getting an average [cadence] of 83 or 85, once 87.”  “[The program was…] very helpful. I am standing straighter, walking faster. My family has noticed. Everyone should do this program.”  “I realized I don’t like cycling too much because of the saddle and it got boring! I don’t like machines in general and prefer to take classes with someone in front who tells me what to do. At the beginning [of the cycling class] the instructors were more engaged and told us what to do and how to go through the exercise and that was fun. Later on they realized everyone was at a different level and people knew what to do and so they didn't engage participants enough.”  “I have noticed despite the cycling my PD does feel like it has progressed slightly. But cycling has helped my freezing. I am signed up for an outdoor 100 mile Parkinson bike ride in the fall.”  “I didn’t think I’d like the program but I really do!”  “I love the cycling program – so much so that I’m willing to drive a great distance to participate.”  “I did the cycling before a couple years ago … so was happy to do it again for the study, but on the whole gym was a little too far to drive for regular classes.”  “I, and people around me have noticed a real improvement in attitude and appearance ever since I started the cycling program. I am continuing high-cadence cycling with enthusiasm.” |
| --- |

^†^Participant comments about the PFP Program were elicited in an unstructured format during mid-study

and eight week-post check-in phone calls. Included comments were chosen to reflect the range of positive and negative feedback elicited. These comments are not intended to represent a quantitative or qualitative analysis of the program but rather to bring the voice of participants into the discussion.
